# Supplementary material for: Morphological Changes in Astrocytes by Self-Oxidation of Dopamine to Polydopamine and Quantification of Dopamine through Multivariate Regression Analysis of Polydopamine Images
Source: Polymers (Basel). 2020 Oct 26;12(11):2483. doi: 10.3390/polym12112483 (PMC7692622; doi:10.3390/polym12112483)
Supplement: Supplementary file 1 [file polymers-12-02483-s001.pdf]

polymers

MDPI

Article

Morphological Changes in Astrocytes by Self-Oxidation of Dopamine to Polydopamine and Quantification of Dopamine through Multivariate Regression Analysis of Polydopamine Images

Anik Karan [1,†], Elnaz Khezerlou [1,2†], Farnaz Rezaei [3], Leon Iasemidis [1,2], Mark A. DeCoster [1,2,4*]

[1] Biomedical Engineering Department, College of Engineering and Science, Louisiana Tech University, Ruston, LA 71270, USA; aka029@latech.edu (A.K.); ekh008@latech.edu (E.K.); leonidas@latech.edu (L.I.)
[2] Center for Biomedical Engineering and Rehabilitation Science, College of Engineering and Science, Louisiana Tech University, Ruston, LA 71270, USA.
[3] Computational Analysis and Modeling Program, College of Engineering and Science, Louisiana Tech University, Ruston, LA 71270, USA; fre004@latech.edu (F.R.)
[4] Institute for Micromanufacturing, College of Engineering and Science, Louisiana Tech University, Ruston, LA 71270, USA.
* Correspondence: decoster@latech.edu

Received: 21 September 2020; Accepted: 21 October 2020; Published: date

Supplementary:

1. Formula Proofs

1.1. Proof of (2)

If Y_i (i = 1,…,n) are functions of m regressor variables $X_i = [x_{i1},…,x_{im}]$ with $n>m$, the multiple linear regression model $Y_i = A{\cdot}X_i$ can be used to linearly approximate the relationship between $\boldsymbol{Y}$ (*DA concentrations*) and $\boldsymbol{X}$ *(RGB color intensities)* [30]. In our application here, $m = 3$ (three colors) and $n = 6$ (six DA concentrations; one of which is for $C = 0$). Also, we had three different trials ($K = 3$) per experiment and the question was how to also incorporate their data in the formulation of the model. Let's denote by $X_i^{(k)}$ (k = 1,…,K) the observation/data point of the ith intensity with the ith concentration from the kth trial. Then the best fitted model over the K trials can be found by minimizing the least squares errors of the trials in some optimal combination that we show below.

We now have a set of multiple linear regression models $\boldsymbol{Y} = A{\cdot}\boldsymbol{X}^{(k)}$. Then, the matrix format of the least squares error function can be expressed as:

$$L = \sum_{k=1}^{K} (\boldsymbol{Y} - A\boldsymbol{X}^{(k)})(\boldsymbol{Y} - A\boldsymbol{X}^{(k)})^T \quad \text{(S-1)}$$

where A is a (1 × m) matrix containing the model coefficients (in this particular application, the intercepts of the model are assumed to be zero, that is, concentration C = 0 if RGB intensities = 0), $\boldsymbol{Y}$ and $\boldsymbol{X}^{(k)}$ are matrices with dimensions (1 × n) and (m × n), respectively. The least squares estimates of the model coefficients must then satisfy:

$$\frac{\partial L}{\partial A} = \sum_{k=1}^{K} 2(AX^{(k)}\{X^{(k)}\}^T - Y\{X^{(k)}\}^T) = 0 \quad \text{(S-2)}$$

which results in:

$$A\sum_{k=1}^{K}\boldsymbol{X}^{(k)}\{\boldsymbol{X}^{(k)}\}^{T}=\boldsymbol{Y}\sum_{k=1}^{K}\{\boldsymbol{X}^{(k)}\}^{T} \quad \text{(S-3)}$$

$$\boldsymbol{Y}=A\left(\sum_{k=1}^{K}\boldsymbol{X}^{(k)}\{\boldsymbol{X}^{(k)}\}^{T}\right)\left(\sum_{k=1}^{K}\{\boldsymbol{X}^{(k)}\}^{T}\right)^{+} \quad \text{(S-4)}$$

Hence, the set of the multiple linear regression models over K trials is reduced to one general regression model of the form $Y=A\bar{\boldsymbol{X}}$, where $\bar{\boldsymbol{X}}=\left(\sum_{k=1}^{K}\boldsymbol{X}^{(k)}\{\boldsymbol{X}^{(k)}\}^{T}\right)\left(\sum_{k=1}^{K}\{\boldsymbol{X}^{(k)}\}^{T}\right)^{+}$ with the superscript ($^{+}$) denoting the Moore-Penrose pseudo-inverse of a matrix [29].

1.2. Proof of (4)

In this case, we consider **X** (the RGB intensities) as a function of **Y** (the DA concentrations) because we are called to estimate **X** if **Y** is known. Then, the simple linear regression model $X_i = B\cdot Y_i$ is used to linearly approximate the relationship between **X** and **Y.** But we still here have data from multiple trials per experiment to fit the model on.

To fit a simple linear regression model to the data over K trials $\boldsymbol{X}^{(k)} = B\cdot\boldsymbol{Y}$ (k = 1,…,K), we have to minimize the least squares errors from each trial. Here, since RGB intensities could be non-zero even if DA concentration is zero, we consider the most general case where the intercept of the fitted line may not be zero. Then, $\boldsymbol{Y}$ is a (2 × n) matrix where all its first-row elements are ones, and B is a (1 × 2) regression coefficient matrix containing the interception and the slope of the fitted line. Then, by defining the squared error function L as:

$$L=\sum_{k=1}^{K}(\boldsymbol{X}^{(k)}-B\boldsymbol{Y})(\boldsymbol{X}^{(k)}-B\boldsymbol{Y})^{T} \quad \text{(S-5)}$$

For minimization of L, the least squares estimators should satisfy:

$$\frac{\partial L}{\partial B}=\sum_{k=1}^{K}2(B\boldsymbol{Y}\boldsymbol{Y}^{T}-\boldsymbol{X}^{(k)}\boldsymbol{Y}^{T})=0 \quad \text{(S-6)}$$

$$B\boldsymbol{Y}\boldsymbol{Y}^{T}=\left(\sum_{k=1}^{K}\boldsymbol{X}^{(k)}\right)\boldsymbol{Y}^{T} \quad \text{(S-7)}$$

$$\left(\sum_{k=1}^{K}\boldsymbol{X}^{(k)}\right)=B\boldsymbol{Y} \quad \text{(S-8)}$$

Hence, the simple linear regression model over K trials is reduced to a simple regression model of the form $\bar{\boldsymbol{X}}=B\boldsymbol{Y}$, where $\bar{\boldsymbol{X}}$ is to be taken as the average of $\boldsymbol{X}$ over the K trials.

2. Figures

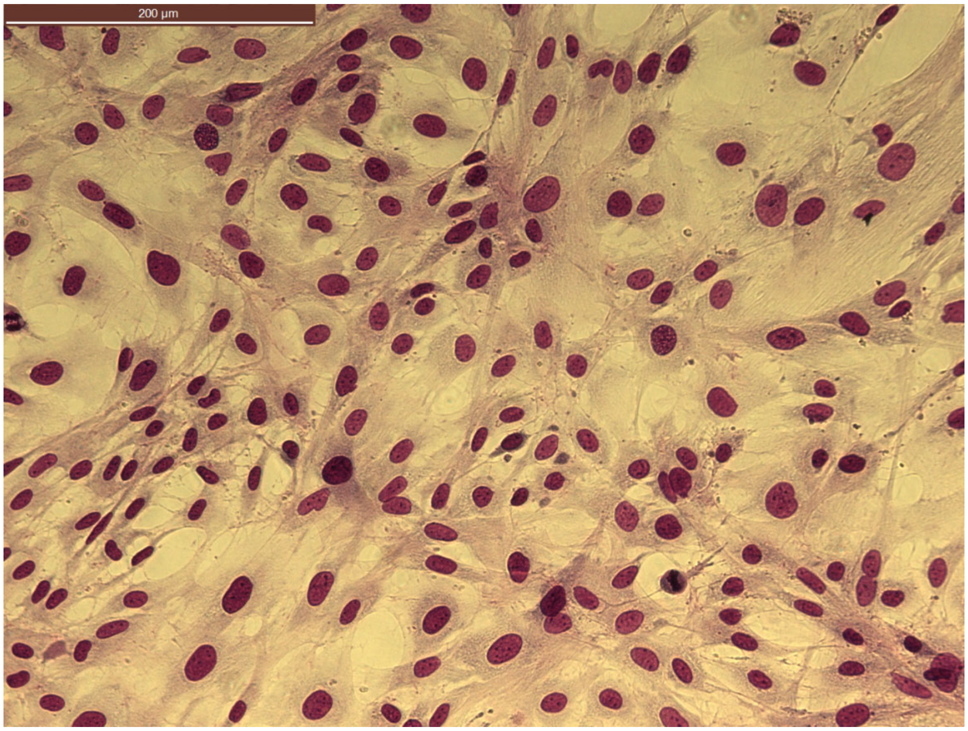

Figure S1: Treatment of 5000 astrocytes/well with no dopamine added (control). Diff-Quik stained astrocytes, 48 h post treatment; scale bar indicates 200 microns.

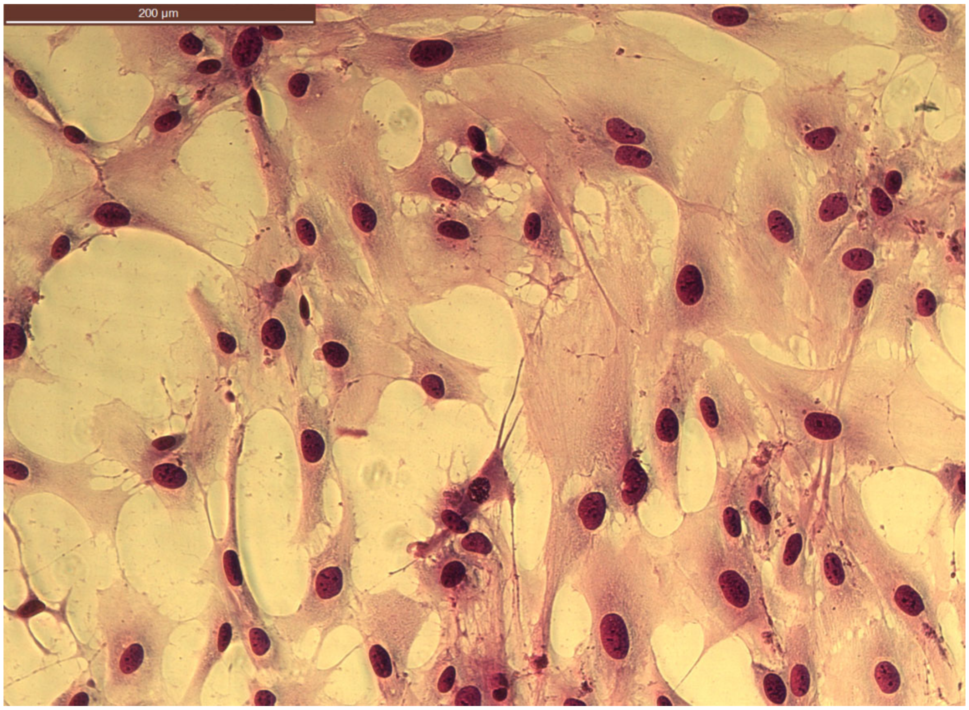

Figure S2: Treatment of 5000 astrocytes/well with 25 µM dopamine. Diff-Quik stained astrocytes, 48 h post treatment; scale bar indicates 200 microns.

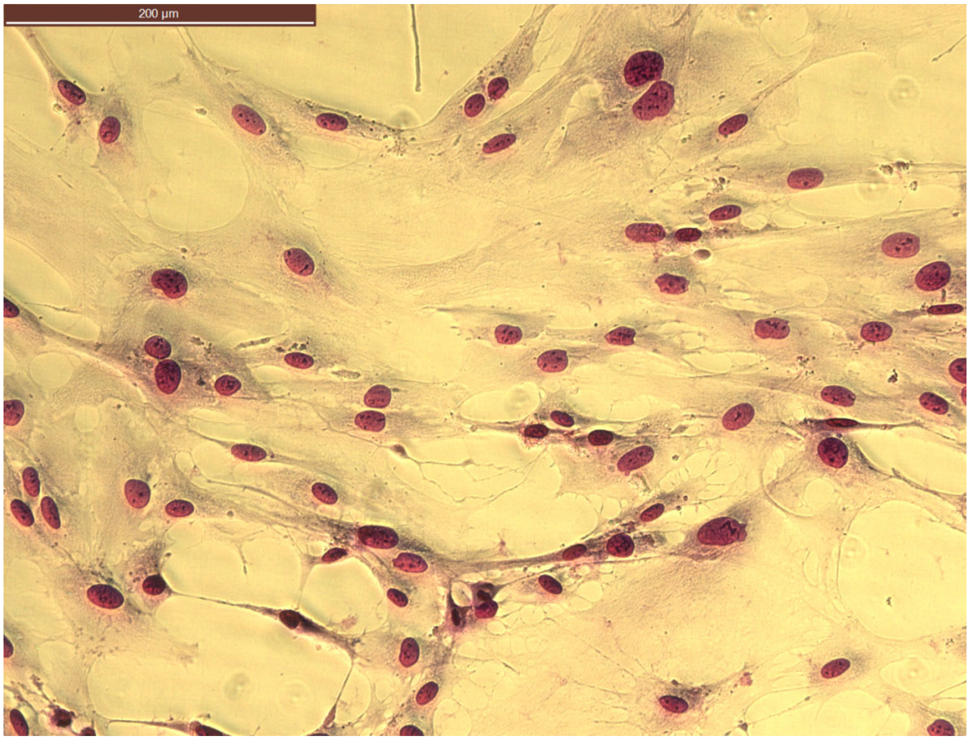

Figure S3: Treatment of 5000 astrocytes/well with 50 µM dopamine. Diff-Quik stained astrocytes, 48 h post treatment; scale bar indicates 200 microns.

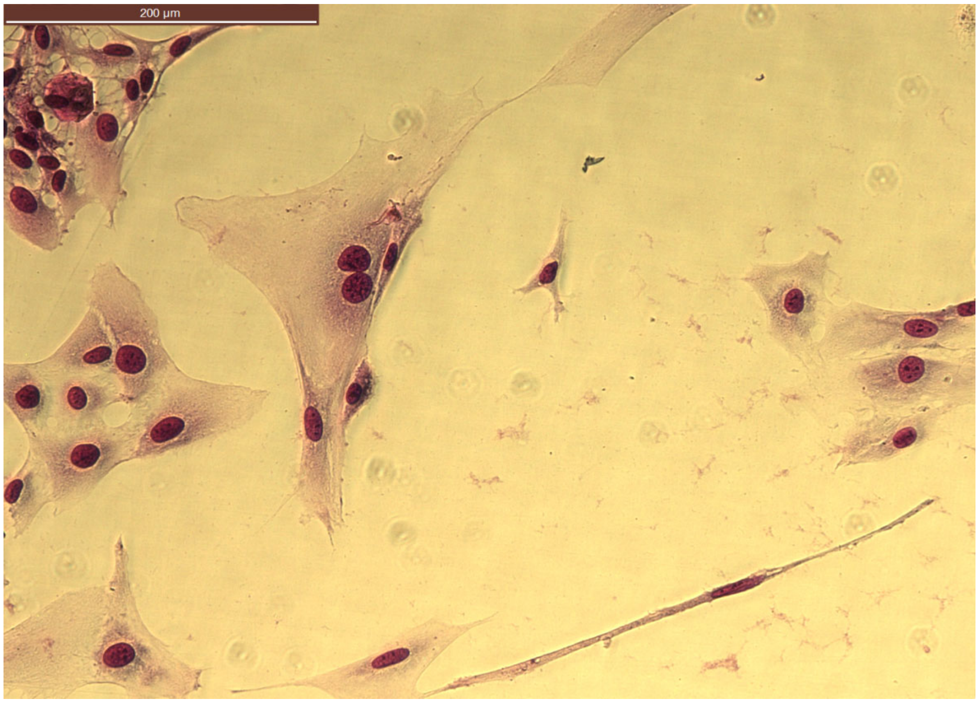

Figure S4: Treatment of 5000 astrocytes/well with 75 µM dopamine. Diff-Quik stained astrocytes, 48 h post treatment; scale bar indicates 200 microns.

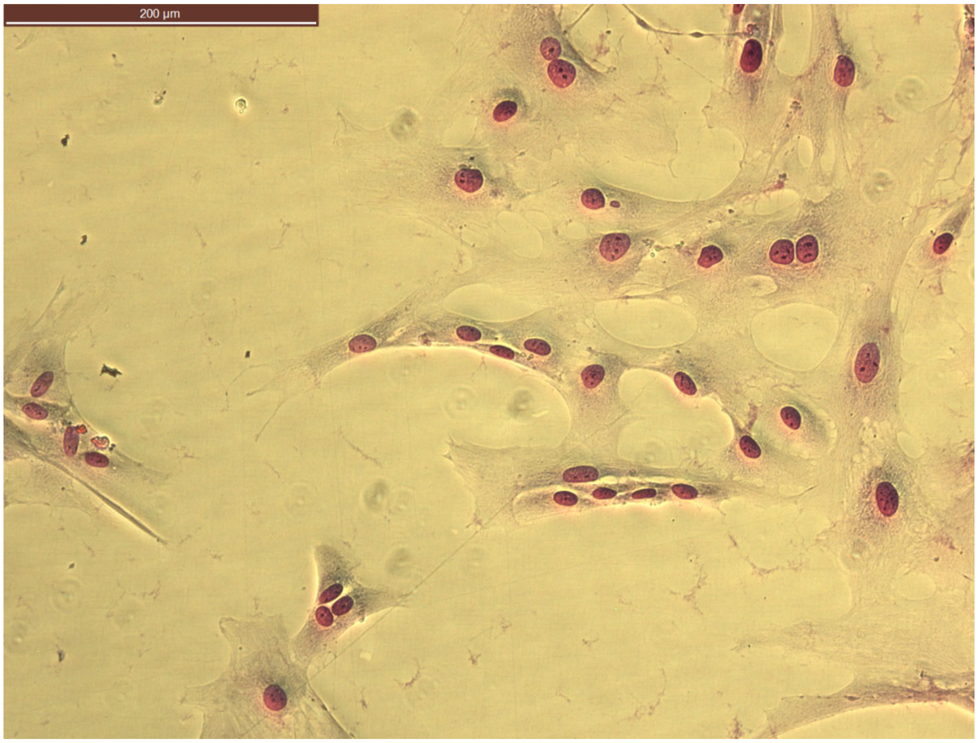

Figure S5: Treatment of 5000 astrocytes/well with 100 µM dopamine. Diff-Quik stained astrocytes, 48 h post treatment; scale bar indicates 200 microns.

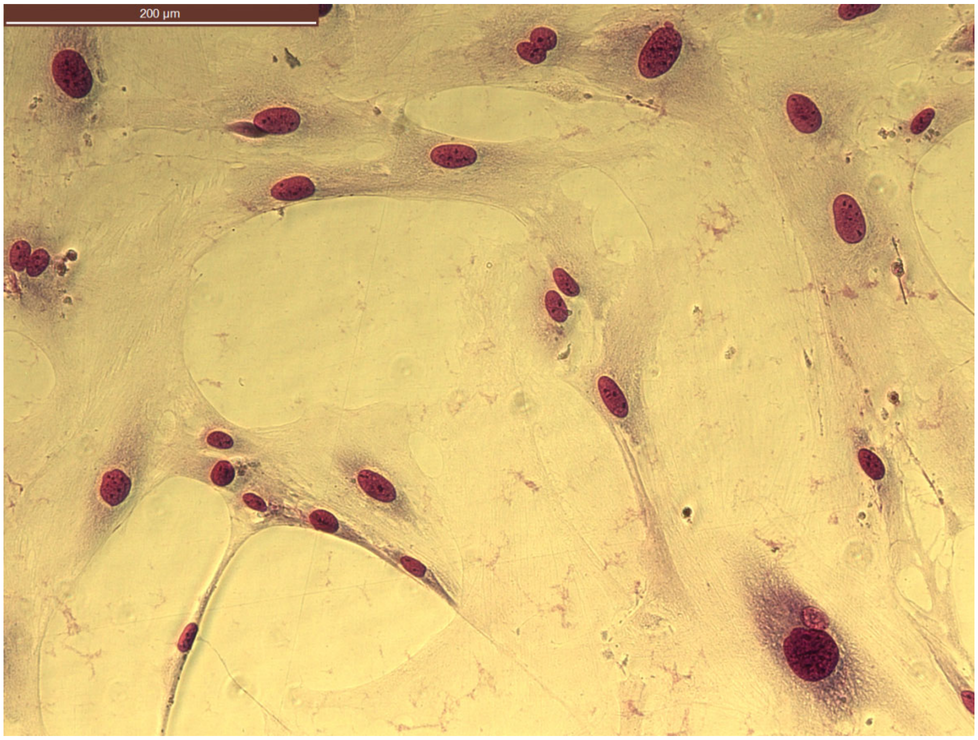

Figure S6: Treatment of 5000 astrocytes/well with 125 µM dopamine. Diff-Quik stained astrocytes, 48 h post treatment; scale bar indicates 200 microns.

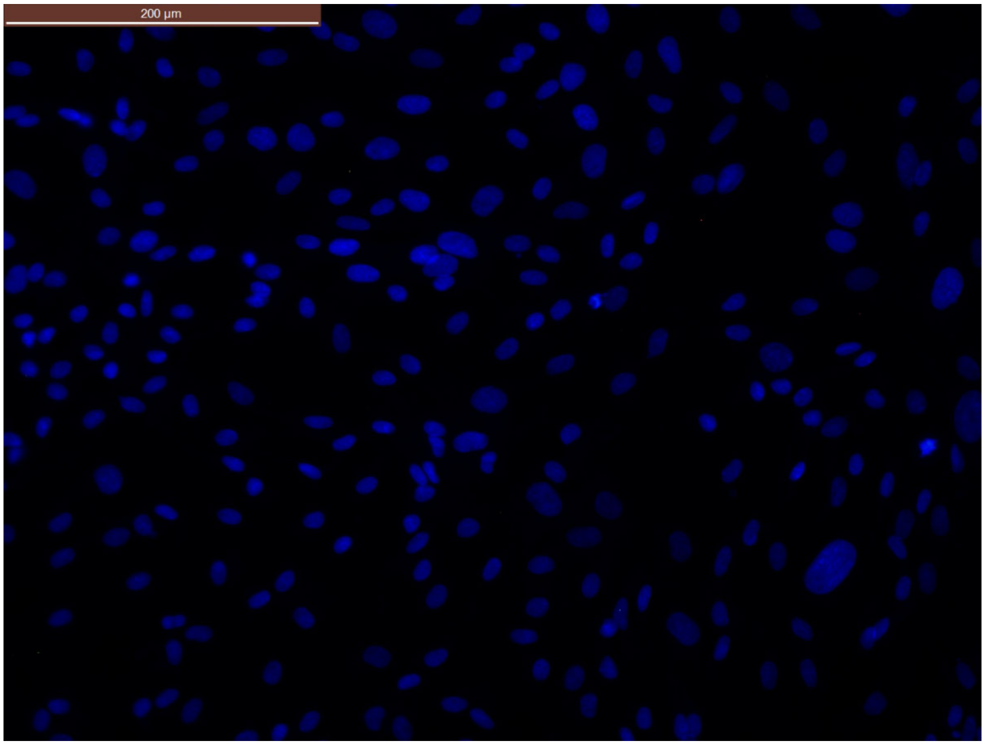

Figure S7: DAPI stained astrocytes (untreated control); scale bar indicates 200 microns.

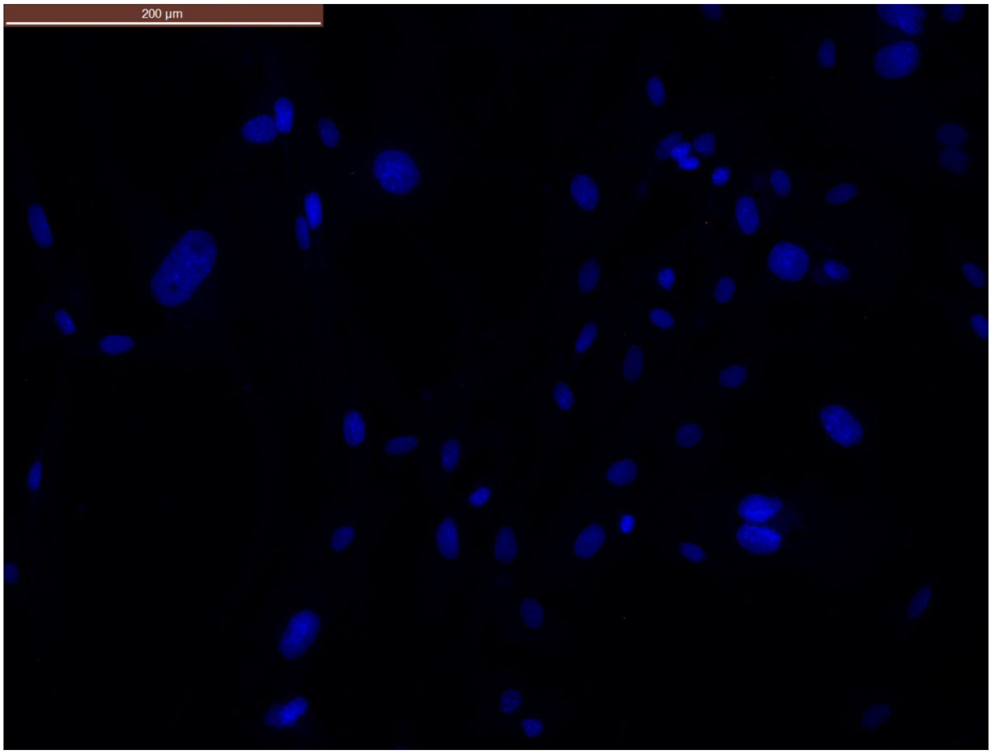

Figure S8: DAPI stained astrocytes (treated with 25 μM dopamine); scale bar indicates 200 microns.

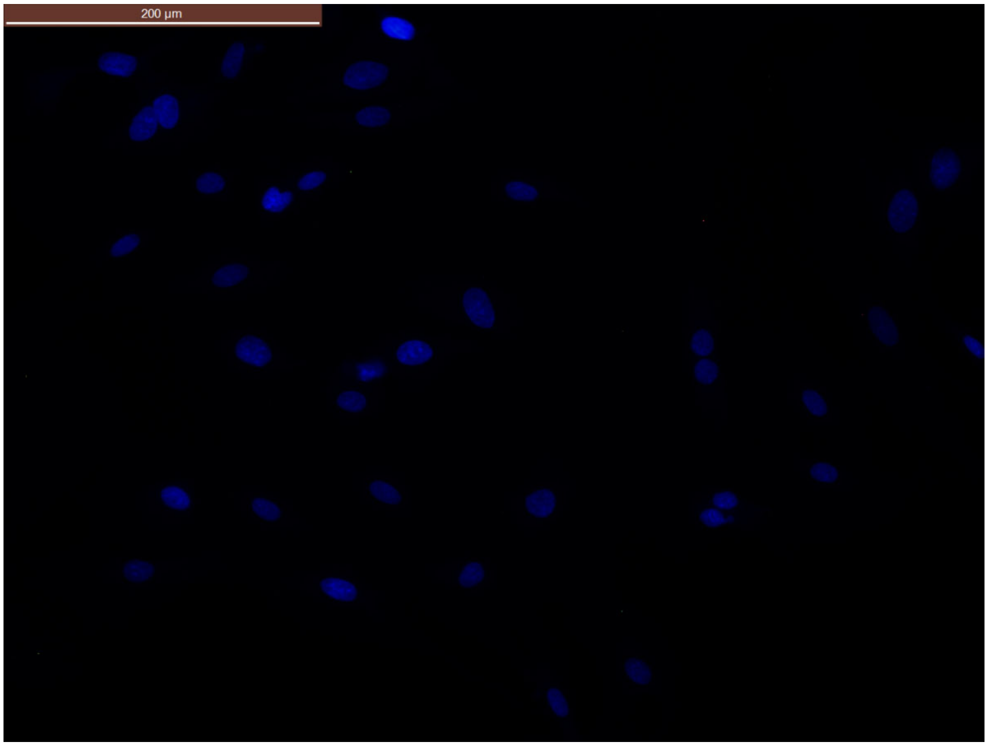

Figure S9: DAPI stained astrocytes (treated with 50 μM dopamine); scale bar indicates 200 microns.

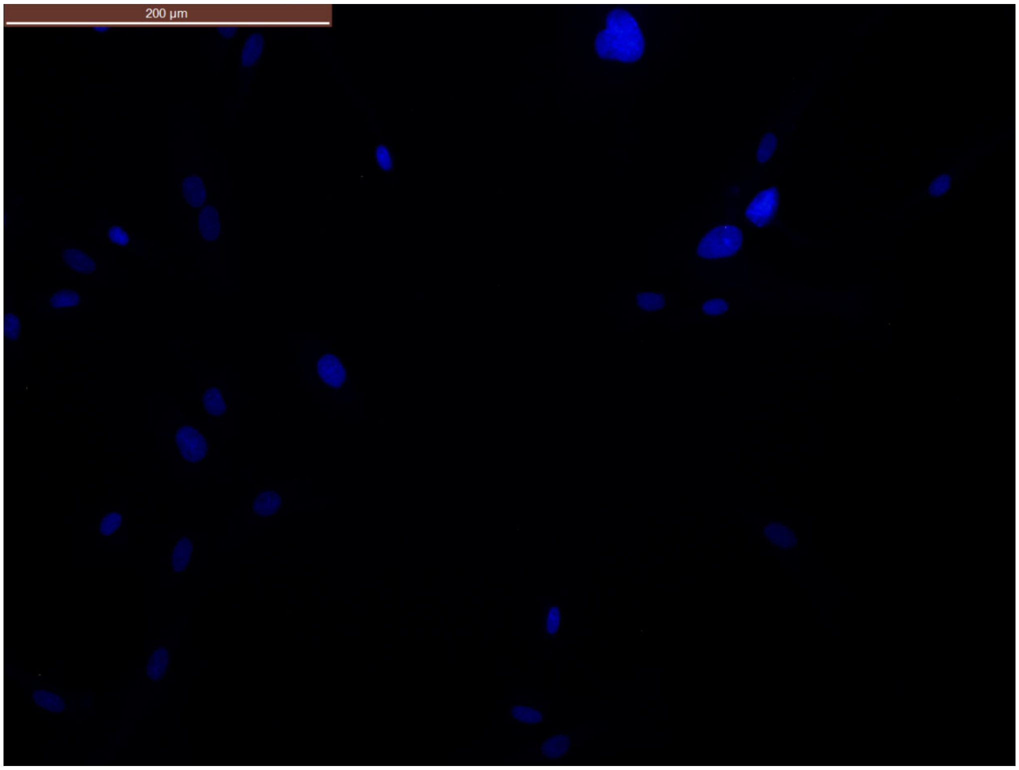

Figure S10: DAPI stained astrocytes (treated with 75 μM dopamine); scale bar indicates 200 microns.

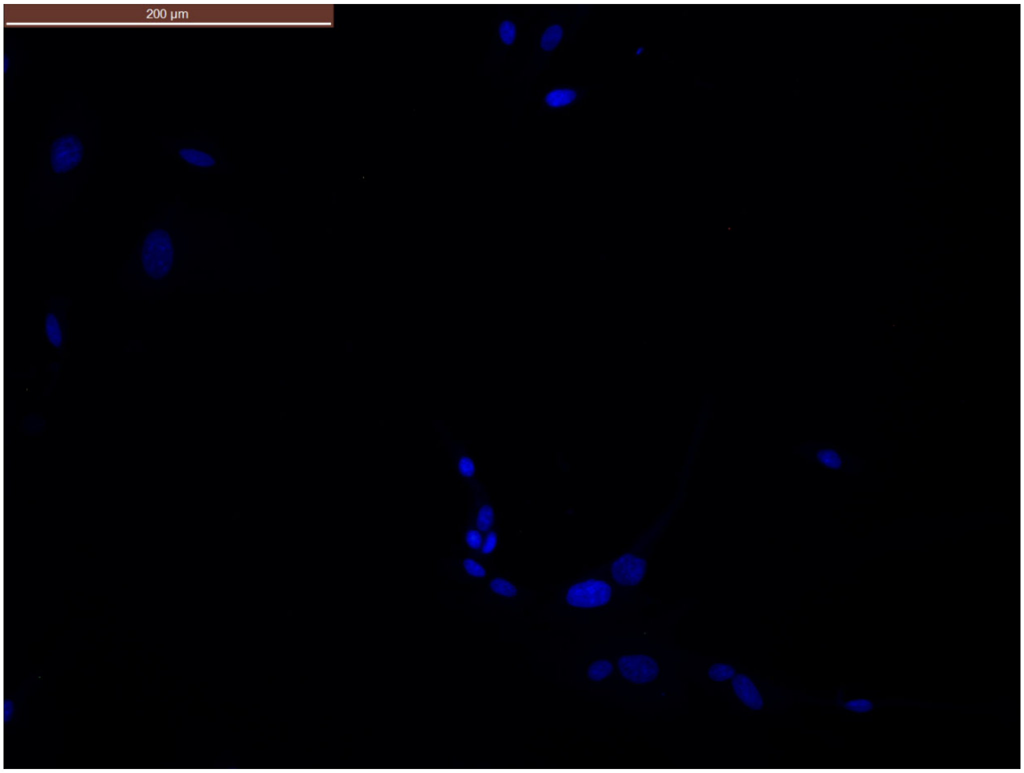

Figure S11: DAPI stained astrocytes (treated with 100 µM dopamine); scale bar indicates 200 microns.

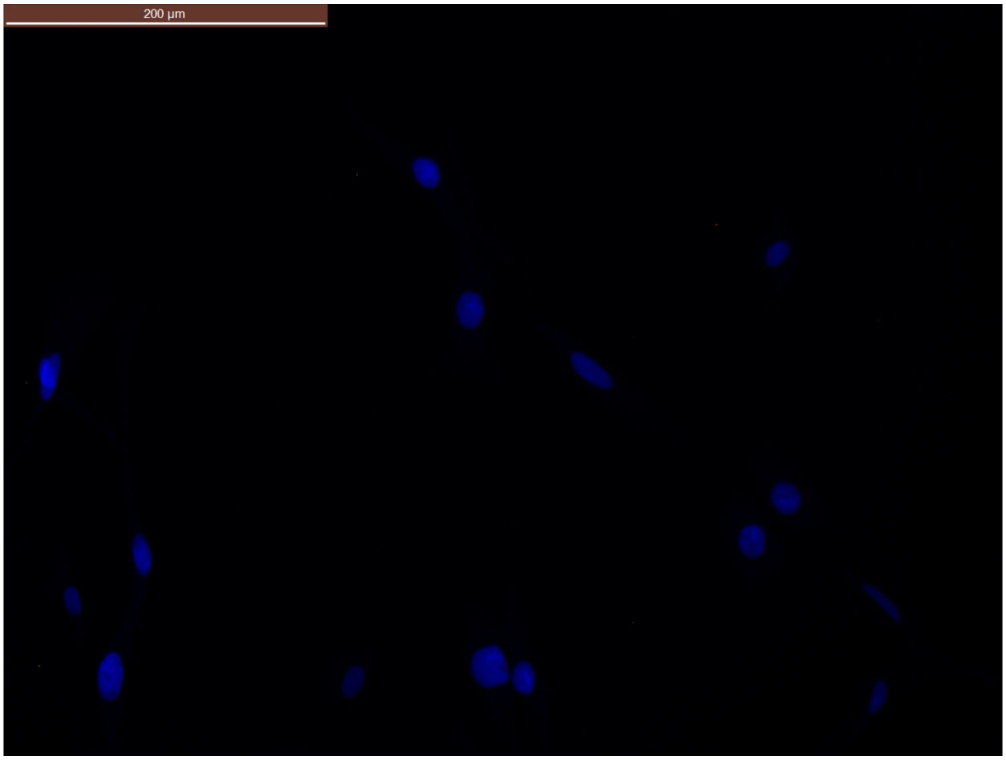

Figure S12: DAPI stained astrocytes (treated with 125 µM dopamine); scale bar indicates 200 microns.
